# Communications

**Published:** 1910-07

**Authors:** William Waugh

**Affiliations:** Professor of Therapeutics, Bennett Medical College, Chicago, Ill.


### COMMUNICATIONS.

---

### PROGRESSIVE MUSCULAR ATROPHY.

---

Editor Journal-Record of Medicin:

In the *Atlanta Journal-Record* a correspondent asks for suggestions on the treatment of a case of progressive muscular atrophy that has been pronounced incurable by very high authorities indeed.

Can any reader of this journal cure it?

The records present a picture characterized by its simplicity —everything has been tried and nothing succeeded. Sometimes the progress of the malady is suspended, but there has been no sufficient reason for attributing the delay to any form of treatment. The way is clear.

Let's go back to first principles: We have a degeneration and death of neural elements, in the cord or periphery. What sickens and kills them? Some destructive agent, parasitic or chemic. The latter almost certainly. How can a chemic poison get to these well-protected structures unless through the circulation, hemic or lymphatic? What can be thus carried to the exceedingly delicate, highly-differentiated nerve cells, that can exert so deleterious an action upon them? The poison of syphilis, gonorrhea, malaria, rheumatism, gout, or other living organism; lead, mercury, arsenic, barium, copper, or other mineral toxin; or more probably because infinitely more common, the toxins from retained and decomposing fecal material.

Here is work for the laboratory—let the bacteriologist ascertain that there is no invading microörganism, animal or plant, at work; let the chemist seek for a mineral intoxining the system; have the urine, blood and feces examined scientifically, for such light as may be afforded—and it is rare that any case occurs where such examinations do not aid the diagnostician. There may be acidosis, or parasites in the alimentary canal; or indicanuria may afford an indication as to the needs. A tapeworm may be imparting a toxic principle to the blood. I have just seen a report in which a man says he has attacks of glycosuria, and always finds intestinal parasites when this occurs. Do you know of any reference to this as a possible cause, in any textbook treating of diabetes? I venture the suggestion that not one of the experts who have examined the case in question ever examined the feces scientifically.

The only ray of hope as to treatment found in the books lies in Gowers' observation of the benefits following the use of strychnine. Leaving the question of whether this may have been due to its power of accelerating intestinal peristalsis and thus removing an unsuspected cause of the malady, there is reason to believe that this potent incitor of vitality may hold in check a morbid process in the neurons, and turn the balance in favor of the recuperative energies. But why stop with strychnine? This is but one of a group of remedies which are now known only to "act like strychnine," but which may and probably do possess individual characteristics peculiar to each. We know for instance that brucine is also an effective local anesthetic, fitting it for certain cases of gastric irritability in which strychnine is unsuitable. How many more such peculiarities might be discovered, were we to make careful examinations of these other agents, such as calabarine, thebaine, laudanine, gelsemine, etc.?

Some years ago a case applied to me, a man who had suffered years before, from hemiplegia. Strychnine had been used until it no longer afforded relief, and when pushed to higher dosage it caused such symptoms that it had to be discontinued. I suggested the use of thebaine, and from it the patient obtained great relief, to his unbounded gratification. This experience seems significant.

I should advise trial of thebaine in the case under consideration, after the indications presented by the thorough examinations urged have been met. Give a milligram of thebaine before each meal, and gradually increase the doses until effects are manifested. Thebaine is not listed by any supply house. I secured mine from Germany, but I presume that any competent pharmacist can extract it from opium for any physician who cares to give it a trial.

Some of these days we will begin to realize what a wealth of valuable agents we are neglecting in our vegetable materia medica, in the alkaloids that are now but curiosities, recognized but never utilized.

William Waugh, M. D.,

*Professor of Therapeutics, Bennett Medical College,*

*Chicago, Ill..*

